# ClimApp—Integrating Personal Factors with Weather Forecasts for Individualised Warning and Guidance on Thermal Stress

**DOI:** 10.3390/ijerph182111317

**Published:** 2021-10-28

**Authors:** B. R. M. Kingma, H. Steenhoff, J. Toftum, H. A. M. Daanen, M. A. Folkerts, N. Gerrett, C. Gao, K. Kuklane, J. Petersson, A. Halder, M. Zuurbier, S. W. Garland, L. Nybo

**Affiliations:** 1 Section for Integrative Physiology, Department of Nutrition, Exercise and Sports, University of Copenhagen, DK-2200 Copenhagen, Denmark; nybo@nexs.ku.dk; 2 TNO, Unit Defence, Safety & Security, Department of Human Performance, Netherlands Organization for Applied Scientific Research, 3769 DE Soesterberg, The Netherlands; 3 International Centre for Indoor Environment and Energy, Department of Civil Engineering, Technical University of Denmark, Building 402, DK-2800 Lyngby, Denmark; henriette.steenhoff@gmail.com (H.S.); jt@byg.dtu.dk (J.T.); 4 Department of Human Movement Sciences, Faculty of Behaviour and Movement Sciences, Vrije Universiteit Amsterdam, Amsterdam Movement Sciences, Van der Boechorststraat 7-9, 1081 BT Amsterdam, The Netherlands; h.a.m.daanen@vu.nl (H.A.M.D.); m.a.folkerts@vu.nl (M.A.F.); n.m.gerrett@vu.nl (N.G.); 5 Thermal Environment Laboratory, Department of Design Sciences, Division of Ergonomics and Aerosol Technology, Faculty of Engineering (LTH), Lund University, 221 00 Lund, Sweden; Chuansi.Gao@design.lth.se (C.G.); kalev.kuklane@ifv.nl (K.K.); jakob.eggeling@design.lth.se (J.P.); amitava.halder@design.lth.se (A.H.); 6 Institute for Safety (IFV), 2718 RP Zoetermeer, The Netherlands; 7 Public Health Services Gelderland Midden, 6828 HZ Arnhem, The Netherlands; moniek.zuurbier@vggm.nl; 8 FritzdorfSport, 218 51 Klagshamn, Sweden; stephen.w.garland@hotmail.com

**Keywords:** heat assessment, cold assessment, mobile application, human thermal models

## Abstract

This paper describes the functional development of the ClimApp tool (available for free on iOS and Android devices), which combines current and 24 h weather forecasting with individual information to offer personalised guidance related to thermal exposure. Heat and cold stress assessments are based on ISO standards and thermal models where environmental settings and personal factors are integrated into the ClimApp index ranging from −4 (extremely cold) to +4 (extremely hot), while a range of −1 and +1 signifies low thermal stress. Advice for individuals or for groups is available, and the user can customise the model input according to their personal situation, including activity level, clothing, body characteristics, heat acclimatisation, indoor or outdoor situation, and geographical location. ClimApp output consists of a weather summary, a brief assessment of the thermal situation, and a thermal stress warning. Advice is provided via infographics and text depending on the user profile. ClimApp is available in 10 languages: English, Danish, Dutch, Swedish, Norwegian, Hellenic (Greek), Italian, German, Spanish and French. The tool also includes a research functionality providing a platform for worker and citizen science projects to collect individual data on physical thermal strain and the experienced thermal strain. The application may therefore improve the translation of heat and cold risk assessments and guidance for subpopulations. ClimApp provides the framework for personalising and downscaling weather reports, alerts and advice at the personal level, based on GPS location and adjustable input of individual factors.

## 1. Introduction

Adverse weather conditions in combination with inappropriate behaviour may have hazardous health consequences. A challenge for the interpretation of the general weather forecast is to weigh the personal situation: activity level, clothing and individual characteristics such as body size all play an important role in heat or cold issues. The ClimApp project (ERA4CS; https://www.lth.se/climapp/, accessed on 20 October 2021) was launched in 2017 with the ambition to combine weather forecasting with personal factors to offer guidance on the assessment of heat and cold exposure and advice for adaptation. To our knowledge, no tool exists that covers the entire range from an extreme cold to extreme heat assessment. ClimApp aims to fill that gap so that weather forecasts are downscaled to the individual and support people in how to dress appropriately and (re-)schedule events. In this paper, we provide an overview of the technical function, extraction of local weather information and integration with individual information constituting the ClimApp tool which is available for iOS and Android devices [1,2,3].

National weather warning systems may come into play when vulnerable groups or groups with prolonged exposure can expect increased safety hazards [4,5]. Obviously, the weather report alone cannot address the unique combinations of the aforementioned variables to assess the risk of thermal issues for the individual. For example, even in arctic conditions heat issues can be experienced during high-intensity exercise with insulative clothing [6,7]. Moreover, long before health issues arise, heat and cold can negatively impact productivity and well-being [8,9]. Both for occupational health and public health, the need for tools to aid individuals in coping with heat and cold situations is increasing [3]. This is especially true for those with a typical long duration of exposure, such as outdoor workers (e.g., construction, agriculture), firefighters, soldiers, and vulnerable populations (i.e., children and seniors).

Internationally established human thermal models and ISO standards and guidelines take the prime factors (environment, activity, clothing) into account for the assessment of thermal stress [10,11,12,13,14,15]. These may be used in existing national heat-health warning systems, but the thresholds used by weather warning systems have large variations between countries [5]. There are also efforts to downscale national thermal stress forecasts to the regional level [5]. For instance, regional occupational heat-health forecasts are accessible via the HEAT-SHIELD platform, a website that combines weather forecasts with specific working conditions and provides a customised long-term heat-health risk assessment based on Wet Bulb Globe Temperature (WBGT) [4]. For national cold-health risk assessments, the windchill index is frequently used, and while other models exist (e.g., UTCI or CoWeDa), their potential is not yet fully used for national weather forecasts [10,13,16]. ClimApp takes the next step by downscaling the weather forecast to the individual level and providing personal cold and heat stress forecasts based on Global Positioning System (GPS) location, personal input and ISO thermal models. Lastly, the tool also includes a research functionality, thus providing a platform for worker and citizen science projects to collect individual data on thermal exposure. In the present paper, the reader will find a detailed description of the models and technical development of the ClimApp and subsequent examples from cold and hot scenarios with associated alerts and advice for the users.

## 2. Materials and Methods

**Figure 1:**
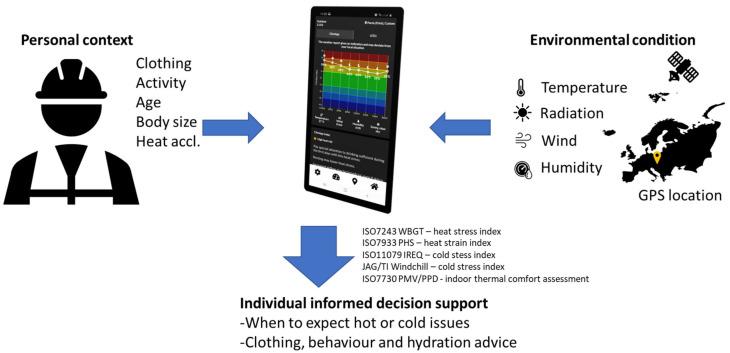
High-level overview of ClimApp: users obtain individualised warning and decision support on heat or cold-related health risks. ClimApp calculates the thermal strain based on established human thermal models (ISO standards) and indices that require input on personal context and environmental conditions. Personal context is entered by the user, and environmental conditions are extracted from local weather data based on GPS location. For indoor environments, the context information is provided by the user.

In this section, the reader will find a comprehensive description of the technical framework and the underlying thermal models of ClimApp. ClimApp supports the following main functionalities (see also Figure 1):

- The device retrieves local current and 24 h weather forecasts based on geographical coordinates.
- The ClimApp index shows the predicted level of heat or cold stress and strain.
- Interpretation and advice of the ClimApp index is presented to the user via icons, graphs and text in the device language.
- The user can customise the model input according to the personal situation, including activity level, clothing, body characteristics, heat acclimatisation, indoor or outdoor situation and geographical location.
- The user can select an individual profile (e.g., themselves or a worker representative), or as a caregiver of children or seniors.

### 2.1. Architecture and Workflow

**Figure 2:**
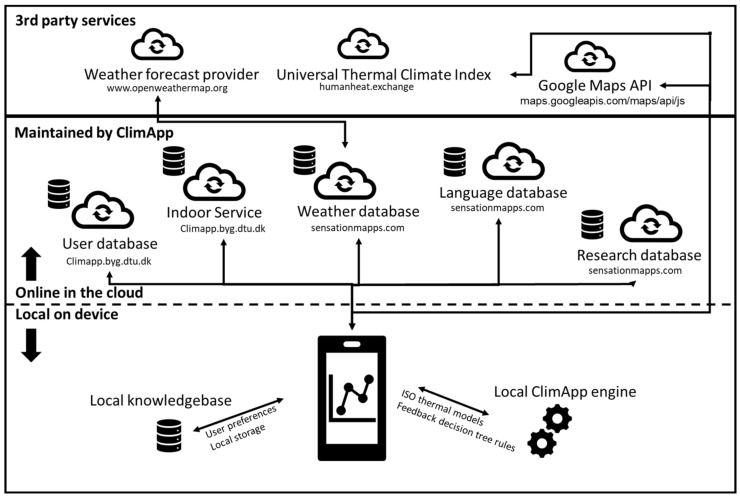
ClimApp architecture overview—see main text for description.

ClimApp is developed on an Apache Cordova open source mobile development framework [17]. From the Cordova framework, a single base of software code is maintained, and each version is consequently built for either the iOS or Android platform. ClimApp source code is publicly available via a GitHub repository [18]. To store and retrieve data, the application communicates with external services (Figure 2). The communication between the services is handled through dedicated application programming interfaces (APIs).

The local device stores user preferences and all data required to run the ClimApp application. ClimApp index calculation, ISO thermal models and advice decision trees run on the local device. User identification is managed through a web service hosted by ClimApp project partners (user database). Local weather is obtained through an intermediate weather database service hosted by ClimApp partners. The intermediate weather database stores each request and prevents overuse of a 3rd party weather forecast provider in case multiple calls to the weather forecast service are made from the same area in the same time frame. Openweathermap.org is used to provide current and 24 h weather forecasts at the time of development of ClimApp. Non-ClimApp thermal index (universal thermal climate index) is obtained through the 3rd party https://humanheat.exchange (accessed on 2 August 2021) API. Custom geographical input is supported through the Google Maps API. Translation files for the supported languages are updated on each use of the app and maintained on the same server as the intermediate weather database. Indoor climate prediction is retrieved from a dedicated Danish Technical University webservice. Research datapoints are stored in a research database if the user gives consent.

**Figure 3:**
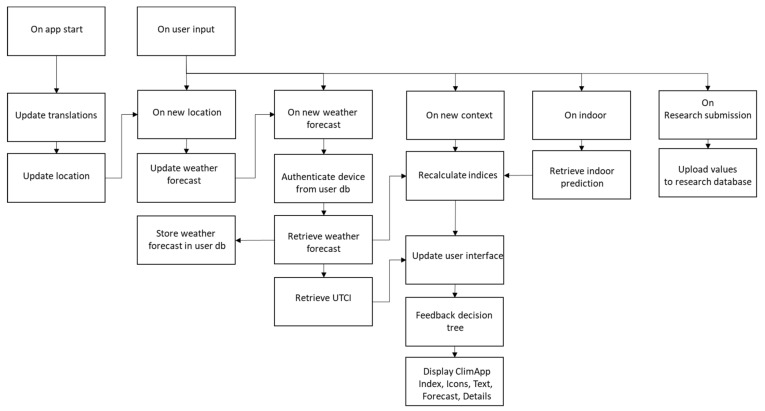
ClimApp workflow—see main text for description.

The information workflow is shown in Figure 3. The main workflow is described as follows: on app start translations are downloaded such that the user can receive text in the user’s local or native language defined in the mobile device. The weather forecast is automatically updated in the app when new GPS coordinates are received, and consequently the thermal indices are recalculated, except for the UTCI which is retrieved from an external source. After calculation of the ClimApp indices, the user interface is updated by evaluation of the feedback decision tree and presentation of the relevant items on the user screen. User interaction can lead to an update of geolocation, weather forecast, context (e.g., activity level, clothing) and consequently an update of the calculations of indices. A user may also participate in research by manually submitting values to the research database.

User data (incl. date and time, geolocation, settings, weather report, model outcomes) are stored in the user database that is only accessible by the DTU research team (see link in ClimApp for privacy policy). On user input, separate subentries of the event queues can be triggered, for instance, if the user selects a custom location the entire event queue from “on new location” is triggered; however, if the user only changes a contextual variable such as the activity level, only the queue following “on new context” is triggered.

### 2.2. ClimApp Environmental Variables

The environmental, activity, clothing and personal inputs that are used by ClimApp are listed in Table 1. ClimApp uses Openweathermap.org as source for current and 24 h weather forecasts (with a 3 h temporal resolution). Openweathermap claims to use a proprietary numerical weather prediction model to reach a spatial resolution of about 500 m. The model uses several data sources including Global NWP models: NOA GFS 0.25 and 0.5 grid sizes, NOAA CFS and ECMWF ERA; weather stations: METAR stations, User’s stations and companies’ stations; weather radar data and satellite data. As not all sites have weather stations, Openweathermap does not provide observational data for the “current” situation but instead uses the model to provide “nowcasts”. The self-reported mean absolute error for nowcasts vs. observation for air temperature is about 0.5 °C, and the root mean squared error is less than 2.0 °C [19]. ClimApp is not dependent on a specific weather data provider, and therefore the remainder of the document refers to weather data providers in general.

Several environmental variables are readily available from standard weather forecasts (e.g., air temperature, relative humidity and wind speed at 10 m altitude). However, these are not sufficient to calculate the thermal indices underlying ClimApp. For instance, water vapour pressure is required to calculate the evaporative driving force between the skin and environment and is derived from air temperature and relative humidity using the Antoine relation (see Table 1 column source).

(1) $$P_{v,air}=0.1\cdot \frac{RH}{100}\cdot e^{\left(18.965-\frac{4030}{T_{air}+235}\right)}$$

Standard weather reports provide wind speed at 10 m height. Except for the windchill index, all other models require wind speed at 2 m from ground level to account for convective and evaporative heat transfer. ClimApp derives windspeed at 2 m according to Liljegren et al. assuming that ground surface is neither radiatively heated or cooled in an urban environment (stability class D) [20].

(2) $$v_{air,2m}=v_{air,10m}{\left(\frac{2}{10}\right)}^{0.25}\approx v_{air,10m}\cdot 0.67$$

For radiative heat transfer, solar radiation is a crucial variable, yet is not included in standard weather reports. Direct and indirect solar radiation is calculated by estimating the angle between the person on the surface of the Earth and the sun based on the geographical coordinates, date and time, as per Liljegren [21]. The total solar radiation (Wm^−2^) is calculated as follows:

(3) $${\overset{\cdot }{Q}}_{solar}=N_{solar,max}\cdot {\overset{\cdot }{Q}}_{solar,toa}$$

With the normalisation of solar radiation before it reaches the surface of the Earth ($N_{solar,max}=0.85)$ and the top of atmospheric solar radiation (${\overset{\cdot }{Q}}_{solar,toa})\;$ calculated as:

(4) $${\overset{\cdot }{Q}}_{solar,toa}=\frac{G_{sc}\cdot \max\left(0,\;cza\right)}{d_{solar}{}^{2}}$$

With the solar constant $G_{sc}$ = 1367 Wm^−2^, the cosine of the solar zenith angle (cza) and distance between sun and earth $d_{solar}$ (A.U.) are calculated as:

(5) $$cza=\cos\left(\left(90{}^{\circ}-e\right)\frac{\pi }{180}\right)$$

(6) $$d_{solar}=1.00014–0.01671\;\cos\alpha -0.00014\cos2\alpha$$

where *e* is the elevation of the sun relative to the surface of the Earth in degrees and α is the mean anomaly of the Earth around the sun, both calculated according to the astronomical almanac [22].

Effects of cloud levels on total radiation are taken into account [23]:

(7) $$\frac{G\left(8\right)}{G\left(0\right)}=1-0.75{\left(N/8\right)}^{3.4}$$

where $\frac{G\left(8\right)}{G\left(0\right)}$ is the transmittance ratio for solar radiation, and N is the cloudiness level in octa. For reference, typical transmittance ratios for cloud overcasts are: cirrus = 0.61, altus = 0.27, cumulus = 0.25 stratus = 0.18 and nimbostratus = 0.16 [23].

For warm conditions, the ClimApp index does not take into account the effect of cloud cover on reduction in direct solar radiation. This provides a worst-case estimation of heat load; that is, in case of intermittent sun exposure (partial clouds), the ClimApp provides advice for the periods that a person is exposed to direct sun light. For cold conditions, the ClimApp index assumes 100% (or 8 octa’s) cloud cover, again to provide a worst-case cold risk assessment.

Black globe temperature (*T_g_*) is calculated from air temperature, wind speed and solar radiation, and natural wet bulb temperature (*T_nwb_*) is calculated from air temperature, air pressure, wind speed, relative humidity and solar radiation according to Liljegren et al. [20]. The wet bulb globe temperature (WBGT) is calculated from air temperature, black globe temperature and natural wet bulb temperature according to ISO 7243 [24].

(8) $$\begin{array}{l} T_{WBGT}=0.7T_{nwb}+0.2T_{g}+0.1T_{air}\\ T_{WBGT,clouds}=0.7T_{nwb,clouds}+0.2T_{g,clouds}+0.1T_{air} \end{array}$$

For the cold model ISO 11079 IREQ and heat model ISO 7933 PHS, radiative heat transfer is incorporated using the mean radiant temperature. ClimApp estimates mean radiant temperature (*T_mr_*) from air temperature, wind speed, and black globe temperature, similar to the approach in the Climate Chip application [25]:

(9) $$T_{mr}=100\cdot {\left({\left(\frac{T_{g}+273}{100}\right)}^{4}+\max\left(0.4\;{\left|T_{g}-T_{air}\right|}^{0.25},\;2.5\;{\left|v_{air,2m}\right|}^{0.6}\right)\left(T_{g}-T_{air}\right)\right)}^{0.25}-273$$

For indoor conditions, air temperature is input by the user, and indoor mean radiant temperature is assumed to be equal to air temperature. To support a user in selecting the right indoor air temperature, a suggested indoor temperature is provided [26]. Based on the suggested indoor air temperature, the suggested relative humidity is calculated as the ratio of actual outdoor water vapour pressure and saturated indoor water vapour pressure:

(10) $$P_{v,air,saturated.indoor}=0.1\cdot e^{\left(18.965-\frac{4030}{T_{air,indoor,suggested}+235}\right)}$$

(11) $$RH_{indoor,\;suggested}=100\%\cdot \frac{P_{v,air}}{P_{v,air,saturated.indoor}}$$

**Table 1:** ClimApp input variables, output variables, dependencies and source of retrieval or derivation.

Type	Input Parameter or Variable	Symbol	Unit	Dependent Model or Output Variable	Source
Environment	Air temperature	T_air_	°C	ISO 11079 IREQ, ISO 7933 PHS, ISO 7243 WBGT, Water vapour pressure, Black globe temperature, Natural wet bulb temperature Mean radiant temperature, Windchill index	Openweathermap.org
	Air temperature indoor	T_air,indoor_	°C	ISO 7730 PMV,	User input
	Atmospheric pressure	P_air_	kPa	ISO 7243 WBGT	Openweathermap.org
	Relative humidity	RH	%	ISO 11079 IREQ, WBGT, Natural wet bulb temperature, Water vapour pressure,	Openweathermap.org
	Relative humidity indoor	RH_indoor_	%	ISO 7730 PMV	Antoine relation [27] using indoor temperature and outdoor water vapour pressure
	Water vapor pressure	P_v,air_	kPa	ISO 7933 PHS, Relative humidity indoor	Antoine relation [27]
	Wind speed at 10 m	V_air,10m_	ms^−1^	Windchill index, Wind speed at 2 m,	Openweathermap.org
	Wind speed at 2 m	V_air,2m_	ms^−1^	ISO 11079 IREQ, ISO 7933 PHS, WBGT, Black globe temperature, Natural wet bulb temperature, Mean radiant temperature	Liljegren et al. [20]—stability class D for rural environments
	Wind speed indoor	V_air,indoor_	ms^−1^	ISO 7730 PMV	Windows closed: V_air,indoor_ = 0.2 ms^−1^ Windows open: V_air,indoor_ = 0.5 ms^−1^
	Cloud cover	N	octa	WBGT cloud	Openweathermap.org
	Rain		ml		Openweathermap.org
	Snow		cm		Openweathermap.org
	Solar radiation	Q_solar_	Wm^−2^	Black globe temperature Natural wet bulb temperature	Liljegren [21]
	Black globe temperature (5 cm)	T_g_	°C	WBGT Mean radiant temperature	Liljegren et al. [20]
	Natural wet bulb temperature	T_nwb_	°C	WBGT	Liljegren et al. [20]
	Mean radiant temperature	T_mr_	°C	ISO 11079 IREQ, ISO 7933 PHS	Ramsey et al. [28]
	Mean radiant temperature indoor	T_mr,indoor_	°C	ISO 7730 PMV	T_mr,indoor_ = T_air,indoor_
	Wet bulb globe temperature	WBGT	°C	ISO 7243 WBGT	Liljegren et al. [20]
	WBGT effective	WBGT_effective_	°C	WBGT effective	ISO 7243 [24]
	Wind chill index	T_windchill_	°C	Windchill index	ISO 11079 [12]
	WBGT reference value	WBGTref	°C	ISO 7243 WBGT	ISO 7243 [24]
	Latitude		°	Solar radiation	GPS or user input
	Longitude		°	Solar radiation	GPS or user input
	Date and time			Solar radiation	Mobile device
Activity	Metabolic rate	M	W	Metabolic heat production	
	Metabolic heat production	Q_met_	W	Recommended Alert Limit	Harris and Benedict [29] ISO 8996 [30], User input
	Metabolic heat production	Q_met_	Wm^−2^	ISO 11079 IREQ, ISO 7933 PHS	Metabolic heat production (W) per body surface area (m^2^)
	External work	W	Wm^−2^	ISO 11079 IREQ, ISO 7933 PHS	W = 0
	Walking speed	v_walk_	ms^−1^	ISO 7933 PHS ISO 11079 IREQ	0.8 m/s—as to approximate air pumping effects in clothing
Clothing	Clothing insulation	I_cl_	m^2^KW^−1^	ISO 11079 IREQ, ISO 7933 PHS	ISO 9920 [31] User input
	Clothing total static evaporative resistance	R_e,T_	m^2^ kPa W^−1^	CAV	ISO 9920 [31]
	Static water vapour permeability index	im_st_	-	ISO 7933 PHS,	ISO 9920 [31] User input
	Air permeability	p_air_	Lm^−2^s^−1^	ISO 11079 IREQ	ISO 9920 [31] User input
	Clothing Adjustment Value	CAV	°C	WBGT effective	Bernard et al. [32]
Person	Age	a	years	Resting metabolic rate	User input
	Height	h	cm	Body surface area, Resting metabolic rate	User input
	Weight	w	kg	Body surface area, Resting metabolic rate	User input
	Sex		-	Resting metabolic rate	User input
	Acclimatisation to heat		-	ISO 7933 PHS, ISO 7243 WBGT	User input
	Body surface area	A	m^2^	Metabolic heat production	du Bois and du Bois [33]
	Position		-	ISO 7933 PHS	standing

### 2.3. ClimApp Index for Assessment of the Thermal Environment

The ClimApp index is a new thermal stress indicator and ranges from −4 to +4, with negative values indicating cold stress and positive values indicating heat stress. In outdoor mode, the determination and interpretation of cold stress is based on ISO 11079 Insulation Required (IREQ) and Windchill [16]. The assessment of heat stress is based on ISO 7243 Wet Bulb Globe Temperature (WBGT) and ISO 7933 Predicted Heat Strain (PHS) [24,34]. In indoor mode, the assessment of the thermal environment is equal to ISO 7730 Predicted Mean Vote (PMV) [35].

#### 2.3.1. Translation of Cold Assessment from ISO 11079 into ClimApp

The ISO 11079 IREQ model calculates the heat balance between the human body and the environment for a given activity level, clothing level and thermal environment. The general heat balance equation is given by [27]:

(12) $$Q_{met}=M-W=K+C+R+E+E_{res}+C_{res}+S$$

where *Q_met_* is metabolic heat production, *M* is the metabolic rate, *W* is external work, *K* is heat transfer through conduction, *C* is heat transfer through convection, *R* is heat transfer through radiation, *E* is heat transfer through evaporation, *E_res_* and *C_res_* are evaporative and convective heat transfer through respiration and *S* denotes heat storage (positive or negative).

The activity level determines the heat production of the body, and the clothing level determines the thermal properties including insulation, water vapour permeability and wind permeability (see Table 1). The thermal environment includes air temperature, relative humidity, air velocity (at 2 m from the ground) and mean radiant temperature (to account for solar radiation). The model output consists of:

- The required whole body clothing insulation to be in thermal balance with the environment to sustain 8 h work (I_cl,neutral_ in Clo);
- The required minimal whole body clothing insulation to allow body cooling to sustain 8 h work (I_cl,minimal_ in Clo);
- The duration limit of exposure—allowing for high cold strain—the value is based on the input clothing level and activity level (DLE_cold_ in minutes).

The IREQ model takes into account the effect of thermoregulatory sweating due to activity for the determination of I_cl,neutral_; however, no thermoregulatory sweating is assumed for the calculation of I_cl,minimal_.

The ClimApp index scales with the worn clothing level (I_cl,user_) vs. I_cl,neutral_ and I_cl,minimal_ is as follows:

- User-specified Clothing level (I_cl,user_) is lower than required minimal clothing level (ClimApp index < −1).
  ◦ ClimApp index = −1 − (I_cl,minimal_ − I_cl,user_);
  ClimApp index = −1.5 means that the user is dressed 0.5 Clo below the minimal required clothing level. The duration limit of exposure will be shorter than 8 h.
- Clothing level is between minimal and neutral (−1 < ClimApp index < 0).
  ◦ Climapp index = −1 + (I_cl,user_ − I_cl,minimal_)/(I_cl,neutral_ − I_cl,minimal_);
  ClimApp index = 0 means that the user is dressed such that no significant cold stress is expected. Workers can continue to work 8 h.
- Clothing level is greater than the neutral clothing level (ClimApp index > 0).
  ◦ ClimApp index = I_cl,user_ − I_cl,neutral_;
  Climapp index = 0.5 means that the user is overdressed by 0.5 Clo and although it may not be a warm environment, heat issues may be expected, for example user may sweat.

The IREQ index indicates whether enough clothing insulation is worn to protect the whole body against cold and does not directly address possible issues with local cooling. Local cooling is covered in the interpretation of the ClimApp index using the windchill index as an auxiliary context variable to indicate risks for exposed skin. The wind chill index is defined as:

(13) $$T_{windchill}=13.12+0.6215\cdot T_{air}-11.37\cdot v_{air,10}^{0.16}\pm 0.3965\cdot T_{air}\cdot v_{air,10}^{0.16}\;[{}^{\circ}C]$$

with air temperature (*T_air_*) in °C and air velocity at 10 m (*v_air_*_,10_) in m/s. The power over air velocity (0.16) is used to scale air velocity at 10 m to air velocity at 2 m from ground level. Advice on the protection of extremities is provided in ClimApp (Cold infographics).

#### 2.3.2. Translation of Heat Stress and Heat Strain Assessment from WBGT ISO 7243 and PHS ISO 7933 into ClimApp

The ClimApp index implements the HEAT-SHIELD platform heat stress assessment algorithm which is based on WBGT ISO 7243 [4,24]. The wet bulb globe temperature (WBGT) is dependent on air temperature, relative humidity, radiative heat and wind speed, which are measured by an air temperature sensor, natural wet bulb temperature sensor and black globe temperature sensor, respectively,–and in part affected by wind speed [24]. In contrast to the heat balance approach of PHS ISO 7933 and IREQ ISO 11079, WBGT ISO 7243 is based on an empirically determined environmental heat stress index and WBGT reference values (*WBGT_ref_*) for which heat stress can be sustained during 8 h of work. The reference values assume that the person is dressed in a long-sleeve cotton shirt and cotton trousers [24]. For other clothing, the WBGT is corrected to the effective WBGT (*WBGT_effective_*) with a clothing adjustment value (CAV).

(14) $$WBGT_{effective}=WBGT+CAV\;[{}^{\circ}C]$$

While WBGT ISO 7243 lists a table with CAV for specific clothing ensembles, ClimApp uses a parametrised equation based on the static evaporative resistance (*R_e_*_,*T*,*s*_) provided by clothing and air [32]:

(15) $$CAV=5.81ln\left(R_{e,T,s}\right)+20.7\;[{}^{\circ}C]$$

Static evaporative resistance of clothing and air is estimated from the total insulation of clothing (*I_T_*: basic clothing insulation + air layer insulation corrected by clothing area factor) according to ISO 9920:

(16) $$R_{e,T,s}=0.16I_{T}=0.16\left(\frac{I_{a}}{f_{cl}}+I_{cl}\right)$$

where *I_cl_* is the basic clothing insulation (m^2^KW^−1^), *I_a_* is the insulation of the air layer of a nude thermal manikin (*I_a_* = 0.085 m^2^KW^−1^) and *f_cl_* is the clothing area factor which accounts for the increase in area for heat exchange due to thickness of the clothing. The clothing area factor is estimated from clothing insulation [31,36]:

(17) $$f_{cl}=1.00+1.81I_{cl}\;\mathrm{for}\;I_{\mathrm{cl}}\;<\;2,$$

(18) $$f_{cl}=1.2424\times I_{cl}^{0.1546}\;\mathrm{for}\;I_{\mathrm{cl}}\;\geq \;2$$

The recommended reference values based on WBGT depend on the activity-level-related metabolic heat production (M in Watts) and heat acclimatisation status of a person. According to ISO WBGT 7243: *“an acclimatised person is one who has been exposed to the hot working conditions (or similar or more extreme conditions) for at least one full working week immediately prior to the assessment period. If this is not the case, the person shall be considered to be unacclimatised.”* [24]. For acclimatised people the *WBGT_ref_* is:

(19) $$WBGT_{ref,acclimatised}=56.7-11.5log_{10}\left(M\right)\;[{}^{\circ}C]$$

For unacclimatised people:

(20) $$WBGT_{ref,unacclimatized}=59.9-14.1log_{10}\left(M\right)\;[{}^{\circ}C]$$

The HEAT-SHIELD Risk Level (HRL) is defined as the percentage of the effective WBGT over the recommended alert limit. The HRL is consequently categorized as not significant (HRL < 80%), low (80% < HRL < 100%), moderate (100% < HRL < 120%) and high risk (HRL > 120%). The broad ranges accommodate interpretation of the heat risk level despite errors that may arise from weather reports, estimated activity level and estimated clothing level. ClimApp follows the HEAT-SHIELD approach for screening heat stress [4]:

(21) $$HRL=\frac{WBGT_{effective}}{WBGT_{ref}}$$

Next, the HRL interpretation is mapped to the ClimApp index as follows:

- HRL less than 0.8 (ClimApp Index < 1).
  ◦ ClimApp index = HRL/0.8;
  ClimApp index = 0.5 means that no significant heat risk is expected.
- HRL between 0.8 and 1.0 and (1 < ClimApp index < 2).
  ◦ ClimApp index = 1 + (HRL − 0.8)/0.2;
  ClimApp index = 1.5 means that the recommended alert limit is being approached and moderate heat stress can be expected.
- HRL between 1.0 and 1.2 (2 < ClimApp index < 3).
  ◦ ClimApp index = 2 + (HRL − 1.0)/0.2;
  ClimApp index = 2.5 means that the recommended alert limit is surpassed and high heat stress can be expected.
- HRL greater than 120% (ClimApp index > 3).
  ◦ ClimApp index = 3 + (HRL − 1.2);
  ClimApp index = 3.5 means that the recommended alert limit is surpassed by more than 20% and severe heat stress can be expected.

Heat stress assessment by the ClimApp index is firstly based on WBGT ISO 7243 and the latter states that it should only be used for screening purposes and that: “*If the values are greater than the reference values, then the risk of heat related disorder increases and it will be necessary to either reduce directly the heat stress or strain at the workplace by appropriate methods or carry out a detailed analysis of the heat stress using ISO 7933*”. The Predicted Heat Strain model (PHS) defined in ISO 7933 provides a method of estimating the thermal strain based on an analysis of the heat exchange between the body and the environment. Similar to the IREQ ISO 11079 model, the PHS ISO 7933 model calculates heat balance between the body and the environment for a given activity level, clothing level and thermal environment. The activity level determines the heat production of the body, and the clothing level determines the thermal insulation and water vapour permeability, but not wind permeability. The thermal environment includes air temperature, absolute humidity, air velocity (at 2 m above the ground) and mean radiant temperature (to account for solar radiation). The PHS and IREQ models take into account the reduction in clothing insulation due to body movement. In ClimApp, slow body movement is assumed for all activity levels, except resting. The PHS model output used by ClimApp consists of:

- The duration limit of exposure before rectal temperature reaches 38 °C (DLE_hot_ in minutes);
- The total amount of sweat loss accumulated over the simulation time (PHS_sweat_ in grams).

#### 2.3.3. Translation of Naturally Ventilated Indoor Thermal Environment Assessment from PMV/PPD ISO 7730 into ClimApp

The Predicted Mean Vote (PMV) and Predicted Percentage Dissatisfied (PPD) models in ISO 7730 are intended to evaluate thermal comfort for indoor environments [35]. The PMV predicts the mean thermal sensation based on a heat balance calculation including activity level, clothing insulation and the environmental parameters air temperature, mean radiant temperature, air velocity, and air humidity. Next the PMV is input to predict the percentage of people that are thermally dissatisfied. For most people (90%) thermally comfortable environments range between −0.5 ≤ PMV ≤ +0.5, cold discomfort is experienced for −2.0 ≤ PMV ≤ −0.5, and warm discomfort is experienced for +0.5 ≤ PMV ≤ +2.0 [37]. For indoor conditions the ClimApp index is equal to the PMV and is consistent with the classes of thermal risk reported in ISO 15265 [37]. That is, ClimApp index < −2 indicates significant risk of cold stress and ClimApp index > 2 indicates significant risk of heat stress; in both cases, action has to be undertaken to mitigate the thermal condition in naturally ventilated indoor environments. Moreover, the overall interpretation of the ClimApp index remains consistent for indoor and outdoor conditions.

### 2.4. ClimApp Personalised Variables

Activity, clothing and body characteristics are customisable for the user to allow downscaling to the end user context. The activity level determines the metabolic heat production that must be dissipated to the environment. ClimApp allows users to select between activity levels in a range from rest to very high activity levels (see Table 2). The associated heat production is derived from ISO 8996, except for the resting metabolic rate, which is estimated according to Harris and Benedict from individual height, weight, age and sex [29]:

(22) $$RMR_{Harris-Benedict,males}=66.4730+5.0033h+13.7516w-6.7550a\;$$

(23) $$RMR_{Harris-Benedict,females}=655.0955+1.8496\;h+9.5634\;w-4.6756a\;\;$$

The Harris and Benedict equation gives resting heat production in kcal per day and is converted to Watts in ClimApp.

Next, the clothing level defines the insulation between a person and the environment. Clothing levels in ClimApp are sampled from ISO7243 WBGT, extended with a winter attire (see Table 3). In ClimApp, users can select a default setting and further fine-tune clothing insulation, vapour permeability and air permeability.

## 3. Results

The methodology of integrating weather forecasts and personal context to provide an interpretation of personal heat and cold stress assessments as described above was implemented in a mobile application that is available on both Android and iOS platforms [1,2]. The main functionalities are distributed over several panels on a single screen: (1) a general overview, (2) a customisation panel, (3) a forecast panel, and (4) a panel with detailed information. Moreover, ClimApp supports the functionality that the geographical location and indoor or outdoor location can be customised. Next, user profile selection (personal, senior caregiver or child caregiver) was used to tune the type of tips to the user. The latter two user profiles may find support in advice adapted to safeguard the elderly or children. Lastly, ClimApp supports a research functionality that allows the app to be used for citizen science projects. The app is available in 10 languages: English, Danish, Dutch, Swedish, Norwegian, Hellenic (Greek), Italian, German, Spanish and French. The app language is set based on the primary language of the mobile device. It works globally.

### 3.1. General Overview Panel

**Figure 4:**
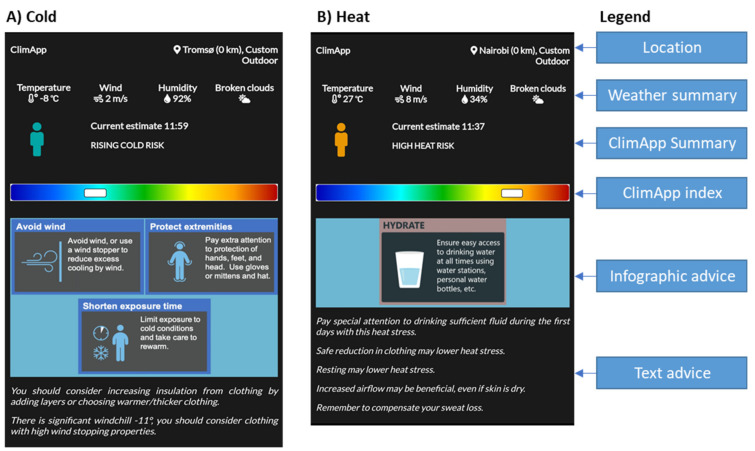
Screenshot of ClimApp summary overview on the current thermal situation (**A**) for a cold scenario in Tromsø (Norway) and (**B**) for a warm scenario in Nairobi (Kenya).

**Figure 5:**
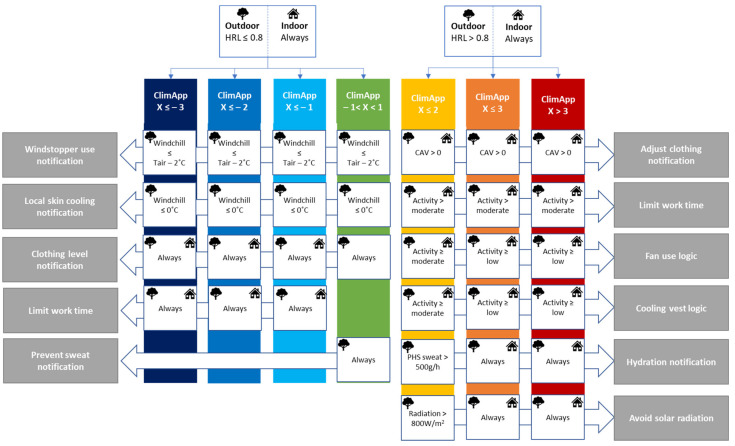
Decision tree logic to derive notifications to user using the ClimApp index, thermal indices and auxiliary variables for indoor and outdoor mode.

The ClimApp main screen with general information is shown in Figure 4. It informs the user of the local weather situation and interpretation, a warning on the severity of the heat or cold stress, and points of advice and attention based on a decision tree (see Figure 5).

For the ClimApp decision tree, the first determination is whether the user should receive notifications to cope with or protect from cold or heat stress. In outdoor conditions a Heat Risk Level (0.8 < HRL < 1.0) indicates the WBGT_effective_ is approaching the WBGT_ref_- (recommended alert limit). For indoor conditions PMV > 1 indicates warm discomfort, and may even indicate heat stress depending on the activity level and clothing level [38]. Suggestions to lower heat stress by clothing adjustment are shown if the clothing level is greater than cotton long sleeves and cotton trousers (CAV > 0). Furthermore, if the activity level exceeds moderate activity the user is made aware that rescheduling work may help to avoid heat stress. Protection and coping strategies by fans and cooling vests are also dependent on the activity level. An extra reminder to focus on rehydration is provided to the user in hot mode.

In cooler environments, ClimApp enters the “cold mode” and notifications may paradoxically point to both cold stress and heat stress at the same time. For instance, if a person is overdressed in a cold (e.g., windchill = −15 °C) environment the ClimApp index will present a positive value, indicating the user can expect to experience heat stress, while considerable local cold stress on exposed skin tissue (e.g., hands) can occur. Clothing with wind stopping ability is advised when the windchill temperature is 2 °C below the air temperature. If the windchill temperature drops below 0 °C, the user is also notified on the potential risk of injury on exposed skin. Clothing level notifications can be directed either to underdressing or overdressing. In cases of underdressing, the user is additionally notified of the duration limit of exposure, and in case of overdressing, the user is urged to prevent sweating.

### 3.2. Customisation Panel

**Figure 6:**
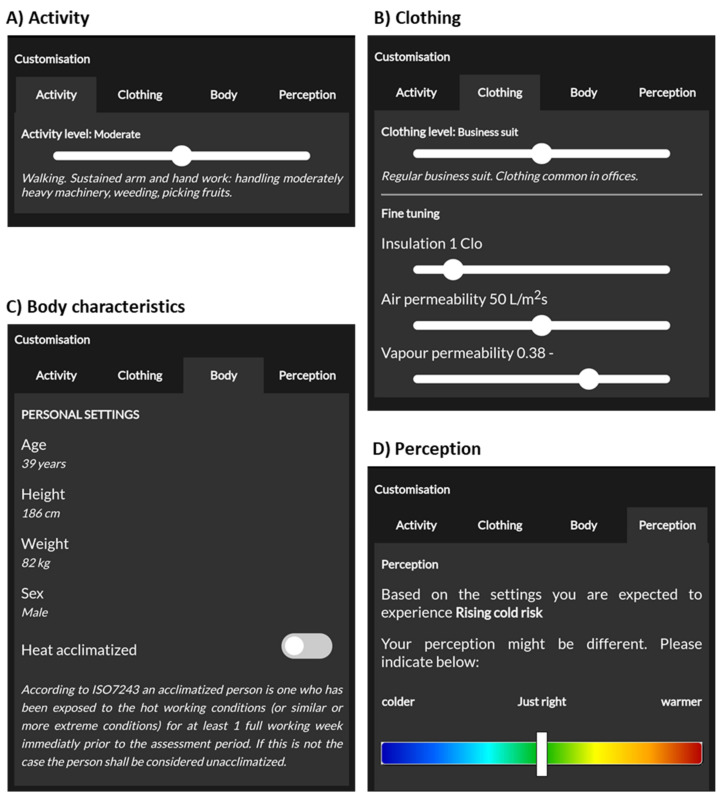
Screenshots of ClimApp customisation facility of (**A**) activity level, (**B**) clothing level, (**C**) body characteristics including heat acclimatisation and (**D**) perception.

The activity level, clothing and body characteristics and acclimatisation to heat are required input parameters for the models underlying the ClimApp index. In ClimApp, these parameters can be set by the user via a customisation panel (see Figure 6). Any change in the context variables triggers a ClimApp workflow (see Figure 3) and causes the ClimApp index to be recalculated. The user is also able to indicate their own perception of the environment. ClimApp uses that information to indicate whether a person might be more or less sensitive to cold or heat and presents that information to the user in the general overview panel. The perception is not used in thermal stress computation as it is not known whether the perception of temperature is related to rational physiological tolerance and exposure limits (see Discussion).

### 3.3. Forecast Panel

**Figure 7:**
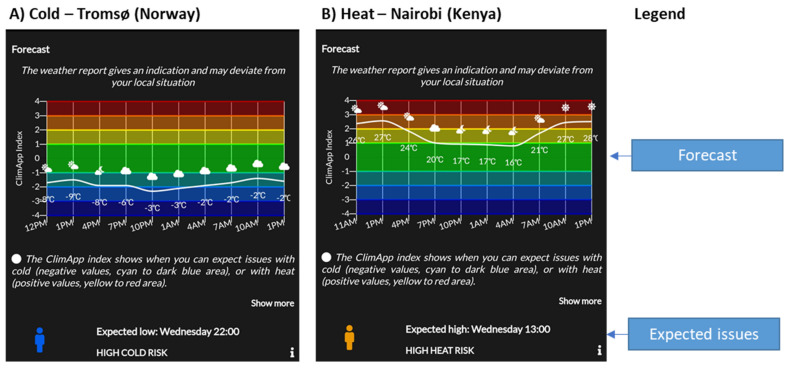
Screenshots of ClimApp 24 h forecast (**A**) for a cold scenario in Tromsø (Norway) and (**B**) for a warm scenario in Nairobi (Kenya). The user is shown a 24 h forecast including icons depicting the weather (e.g., sunny, cloudy, rain and air temperature) as well as a specific notification on what time issues are expected. Note that in Figure 7A the highest cold strain (10 p.m.) does not occur at the lowest air temperature (−3 °C at 10 p.m. vs. −9 °C at 1 p.m.); this is a direct result that the ClimApp index takes into account all six basic factors including the effect of wind and solar radiation on the actual cold stress and not only air temperature.

The forecast panel shows the progression of the ClimApp index for the upcoming 24 h given the chosen activity and clothing level (see Figure 7). Specific attention is paid to time periods where high heat or cold issues are expected, i.e., the ClimApp index dips below −2 or exceeds +2. The forecast panel also includes a tab that directs users to a UTCI 24 h-index forecast (for simplicity not shown in Figure 7, but available for users interested in using this thermal stress index as an alternative or addition to the ClimApp index).

### 3.4. Detailed Information Panel

**Figure 8:**
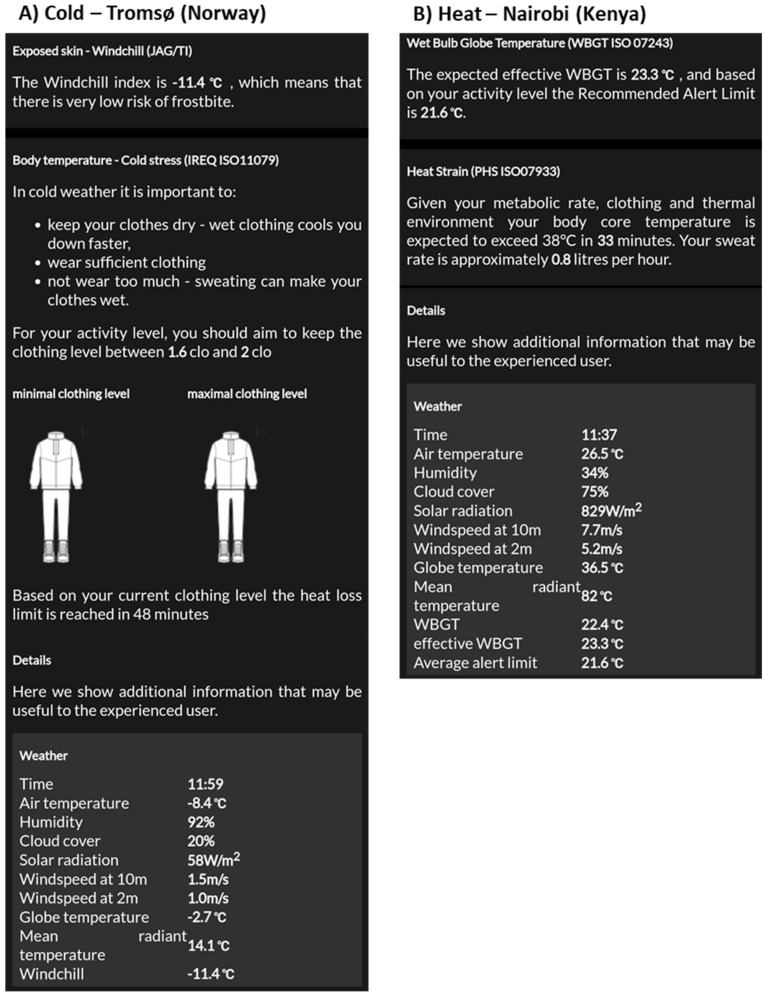
Screenshot of ClimApp detailed information panel with current weather data. (**A**) for a cold scenario in Tromsø (Norway) the user is shown an interpretation of the windchill index and specific information based on ISO 11079 on advised clothing level and duration limit of exposure given the current clothing as input by the user and (**B**) for a warm scenario in Nairobi (Kenya) the user is shown an interpretation of the WBGT ISO 743 as well as a duration limit of exposure and expected sweat losses according to PHS ISO 7933. Furthermore, for both situations, detailed weather data are shown.

Details of the underlying independent model outcomes are presented depending on the decision tree interpretation regarding whether heat or cold stress is expected (see Figure 8). In cold conditions the windchill index provides information on the risk of freezing of exposed skin, and ISO 11079 provides information on required clothing information and the expected duration limit of cold exposure. For warm conditions the calculated WBGT is presented as well as the recommended alert limit. Furthermore, PHS (ISO 7933) provides information on how long it takes before the body core temperature exceeds 38.0 °C and the expected sweat loss per hour. Moreover, weather values are presented in the detailed panel that are required to calculate the models underlying the ClimApp index.

### 3.5. Location Panel

**Figure 9:**
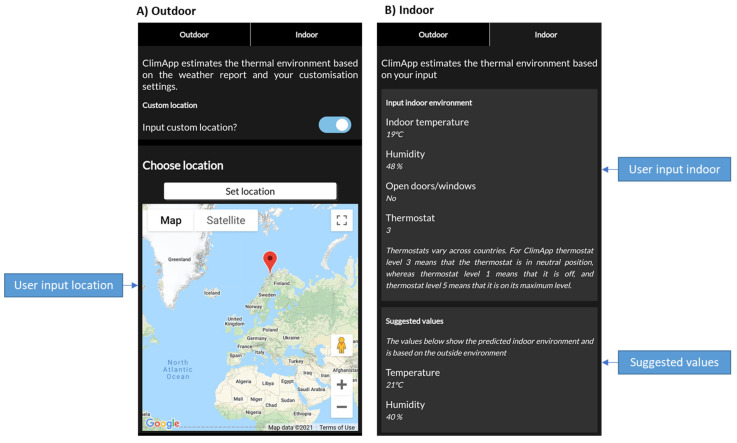
Screenshot of ClimApp location selection panels. (**A**) For outdoor conditions, the ClimApp index is based on the local weather report. Local weather reports are obtained from either GPS coordinates from the mobile device, or user input on a Google Map interface. (**B**) For indoor conditions the ClimApp index is based on user input. The user is provided with suggested values based on the weather report, window state and thermostat setting.

By default, ClimApp uses the mobile GPS coordinates to retrieve the weather report. With the location panel the user can choose a custom location using a Google Map interface (see Figure 9A). There is no limitation on the location chosen by the user; it works globally. In case the location of the user changes, the ClimApp index is refreshed according to the workflow scheme in Figure 3.

For indoor conditions, the user can define the indoor temperature and indoor humidity, indicate whether windows are open or closed and define a generalised thermostat setting between 1 and 5 (see Figure 9B). Thermostats vary across countries, and for ClimApp thermostat level 3 means a neutral position, 1 means that the heating is off and thermostat 5 means that the maximal heating is applied. To support the user in selecting an indoor temperature, a suggested indoor air temperature and the corresponding relative humidity are presented, yet only the values input by the user are used. The algorithm to determine indoor temperature is dominated by Mediterranean climates where indoor settings relied mostly on natural ventilation cooling to maintain the indoor temperature [26].

### 3.6. Profile Panel

**Figure 10:**
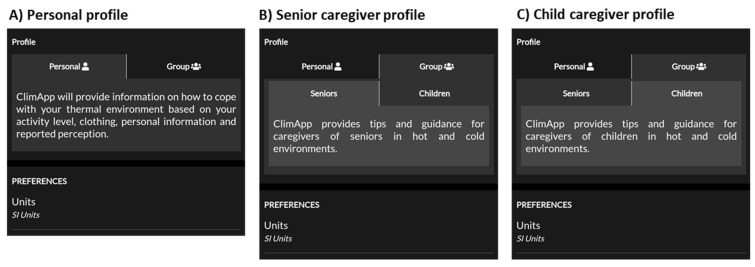
Screenshot of ClimApp profile selection panel. (**A**) A personal profile is the default setting in ClimApp and tips and advice are based on occupational health guidelines. (**B**) Selecting the senior caregiver profile will cause ClimApp to provide feedback and recommendations that are relevant for senior caregivers. (**C**) Selecting the child caregiver profile will cause ClimApp to provide feedback and recommendations that are relevant for childcare providers.

**Figure 11:**
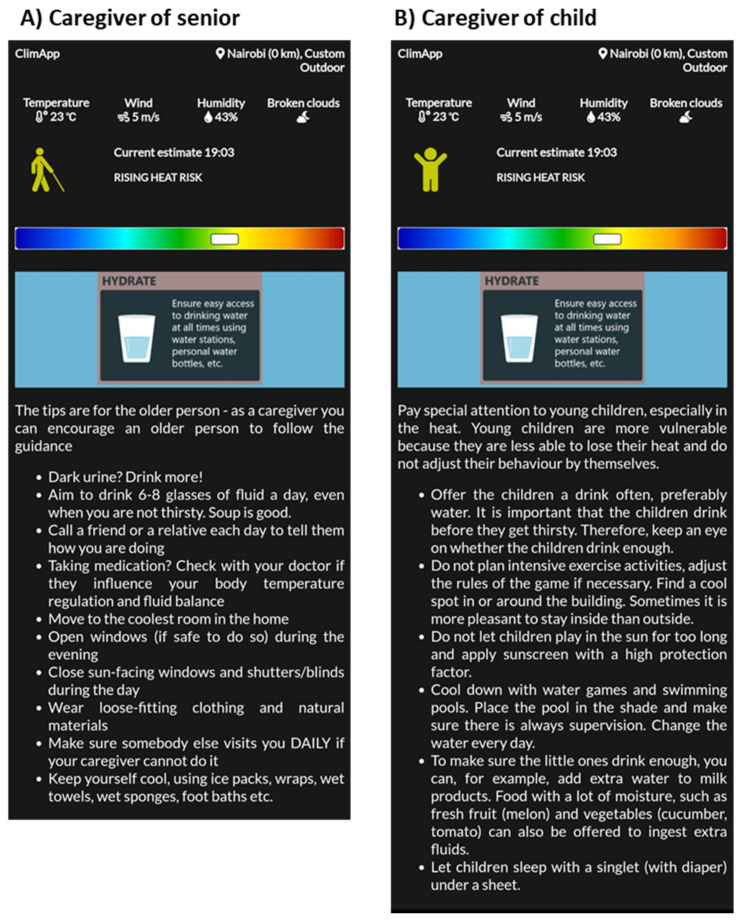
Screenshot of ClimApp general information panel with specific heat mitigation tips for (**A**) caregiver to senior person and (**B**) caregiver to children.

ClimApp supports a personal profile, a senior caregiver profile and a childcare provider profile (see Figure 10). The profile does not influence the calculation of the ClimApp index, yet it does influence the advice that is given to the user such that relevant information for the specific group is shown (see Figure 11). Only profile-specific information for warm conditions is provided, as there seems to be a gap in cold information specifically aimed at children and the elderly.

### 3.7. Research Panel

**Figure 12:**
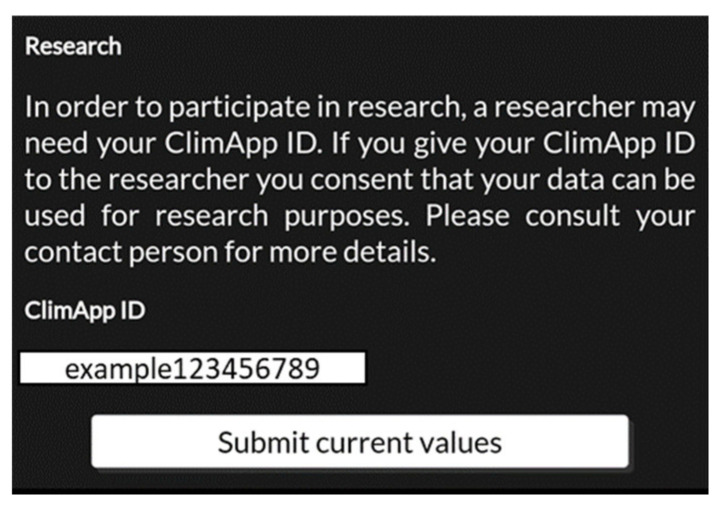
Screenshot of ClimApp research functionality which allows users to participate in citizen science programmes if the users consent.

The research panel allows users to participate in citizen science, and societal applied science projects (see Figure 12). ClimApp supports momentary assessments of the local thermal environment, including metadata (date, time and geolocation), the activity level, clothing level, body characteristics and perceived thermal sensation of users, as well as the ClimApp index itself and outcomes of all models underlying the ClimApp index. The research functionality is not automated and requires an explicit consent and action by the user to submit current values. The data are stored in the research database. There is no public access point to the data. Researchers can only request data for unique ClimApp IDs by contacting the ClimApp consortium.

## 4. Discussion

ClimApp integrates and makes four international standards (WBGT, PHS, PMV/PPD and IREQ) of human thermal environments accessible and usable for all mobile phone users. It helps individual and organisational users get timely warnings and recommendations to cope with extreme heat and cold events:

- **Heat and cold stress assessments and warnings** based on ISO human thermal models and indices presented as a single integrated index ranging from −4 (extremely cold) to +4 (extremely hot), where a value between −1 and +1 is associated with low thermal stress.
- **Individualised input:** activity level, clothing level, body characteristics, heat acclimatisation, indoor/outdoor situation and geographical location
- **Profile-based output**: individual worker, caregiver of children, or caregiver of elderly.
- **Current and forecast weather:** heat and cold stress warning, advice for adaptation such as clothing level, exposure reduction, and hydration in the form of infographics and text.
- **10 languages**: English, Danish, Dutch, Swedish, Norwegian, Hellenic (Greek), Italian, German, Spanish and French.
- **Research functionality**, to collect individual data on thermal exposure and subjective response for worker and citizen science projects.

### 4.1. Estimating Heat Risk Level

**Figure 13:**
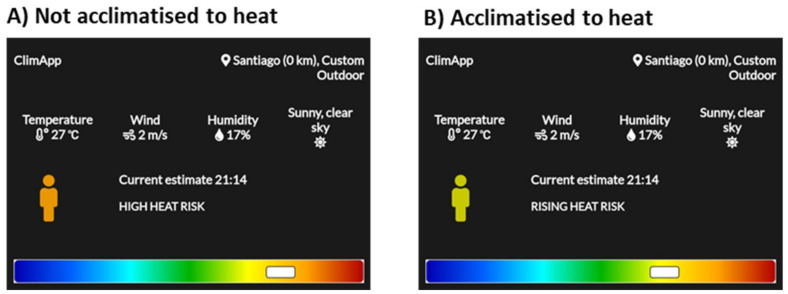
ClimApp case where there is a mixed work force, including (**A**) people not acclimatised to heat and (**B**) people acclimatised to heat.

Estimating the effect of extreme weather events, activity level, clothing level or heat acclimatisation on heat risk level is a major use for ClimApp. For instance, a mixed workforce may consist of people that are more or less acclimatised to the hot working conditions and consequently exposed to a different level of heat risk (Figure 13). ClimApp can assist in deciding for what part(s) of the workforce extra heat mitigation is required, or if all parts of the workforce fall into the same heat risk category, they should all be treated equally.

**Figure 14:**
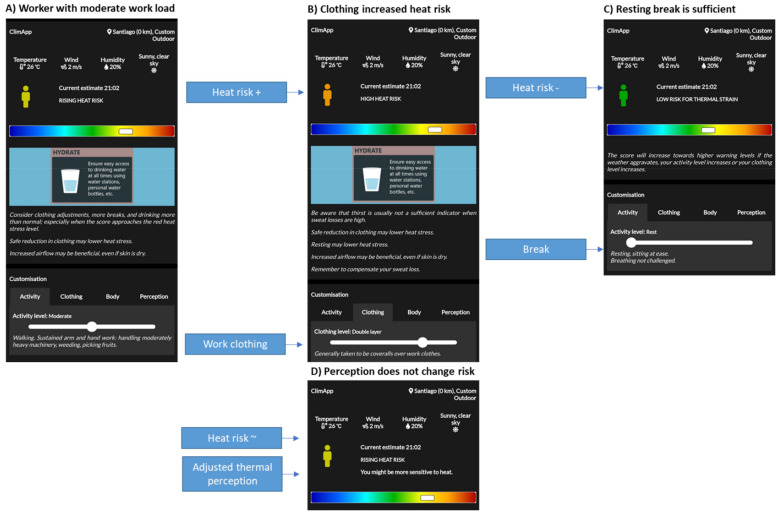
ClimApp case for (**A**) a worker with a moderate workload working in warm dry conditions, (**B**) the same worker wearing double-layered clothing and then (**C**) taking a break. (**D**) If a user feels warmer or colder and inputs that via the perception functionality, ClimApp will inform the user they may be more or less sensitive, although this will not affect the calculated/estimated risk level.

Another example is for a worker with a moderate workload working in warm dry conditions. The activity level causes a rising heat risk for the individual (Figure 14A). However, the required work clothing effectively increases the heat risk level to a high heat risk and requires mitigation (Figure 14B). The warm environment can be fully compensated by rest breaks, and no additional fans are required (Figure 14C). The worker may report feeling less warm, or warmer than the risk level predicted by ClimApp. This will not influence the rational estimate of the risk level, but it will show on the dashboard that the worker may be more or less sensitive to heat (Figure 14D).

### 4.2. Estimating Cold Risk Level

**Figure 15:**
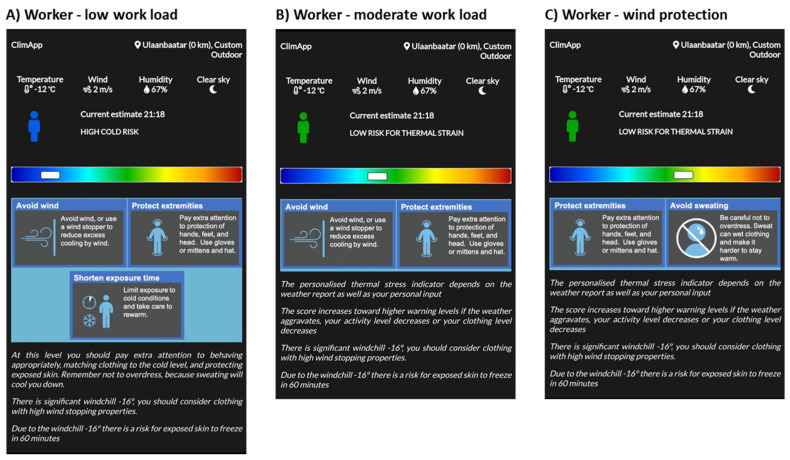
ClimApp case for cold conditions for (**A**) a worker with a low workload, (**B**) a worker with a moderate workload, and (**C**) a worker with a moderate workload and wind protection.

Estimating cold risk and deciding on the mitigation measures to use is mainly a balancing act between activity level and clothing level, weather conditions and exposure time. For instance, a worker with a low workload may experience a high cold risk at −12 °C (Figure 15A). ClimApp can also inform the user that there is also a high wind chill, which is a major cause of excess heat loss, and at the given temperature (−16 °C) may even cause freezing of exposed skin. Increasing the workload to a moderate intensity lowers the overall cold risk, yet it is not a solution to the wind chill (Figure 15B). Avoiding wind (e.g., finding shelter) or adding wind protection (lowering the air permeability of the clothing ensemble) may effectively mitigate cold risk (except the risk of freezing for exposed skin), and even may cause overdressing (Figure 15C). The worker may have to adapt the wind protection and limit cold exposure time during work to maintain a safe working environment.

### 4.3. User Evaluation and Sensitivity Analysis

**Figure 16:**
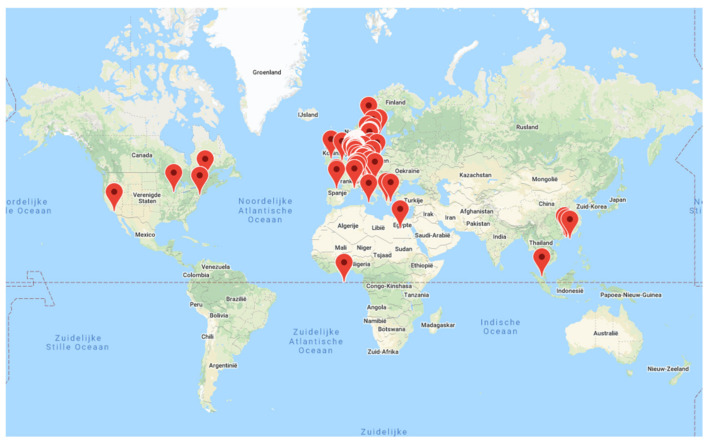
Registered locations (n = 1745) in the research database which is used for user evaluation. Map is created with JS Google Maps API v3.

A ClimApp user evaluation was conducted using the research functionality in hot and cold conditions (n = 1745, see also Figure 16). For the hot environment user evaluation by caregivers of children (summer camp), elderly and young adult evaluations were carried out during a study in summer 2020. The user evaluation consisted of analyses of deviation of ClimApp heat stress prediction vs. subjective heat perception, relevance of tips from the app and app usability. For the cold user evaluation, two usability studies were performed in 2020 and 2021, where improvements from the first usability study were implemented in ClimApp and consequently tested in the second usability study. Furthermore, a field study on the validation of cold stress prediction by ClimApp was performed in the cold with 38 users. A preliminary analysis and comparison of the predicted cold stress by ClimApp and user perceived thermal sensation in the cold showed that the mean predicted ClimApp index score (0.44) is consistent with the mean perceived thermal sensation score (0.34) when the users personalised and adapted their activities and clothing levels to the cold. The evaluation methodologies, results and interpretations are presented in separate papers and have been submitted and are currently under review.

**Figure 17:**
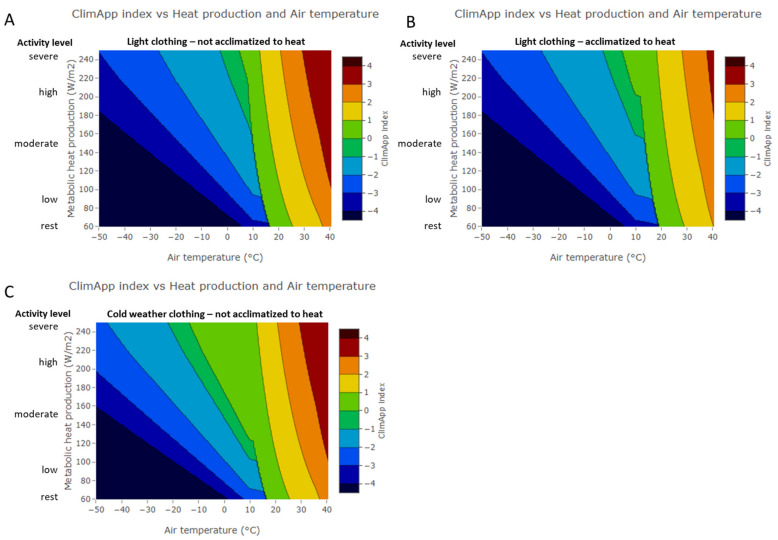
Sensitivity analysis of ClimApp index for meteorological input (air temperature) and personal input (activity level). Panel (**A**): dressed in light clothing, non-acclimatised to heat; panel (**B**): dressed in light clothing, acclimatized to heat; panel (**C**): dressed in cold weather clothing and not acclimatised to heat.

In general terms, the validity of ClimApp with respect to heat and cold risk assessments is equal to the ISO standards that underly its calculation. However, ClimApp may be sensitive to error in the input by either the weather report, user input on activity, clothing or acclimatisation status. The effect of variation on input was made clear with a sensitivity analysis of the ClimApp index for varying air temperature and activity level as shown in Figure 17. For the analysis, the vapour pressure was kept constant (equal to 20C, 50% rh) for all temperatures (yet relative humidity was capped at 100% for low air temperatures), wind speed was set at 1 m/s, and no solar irradiation was present. The person was set at 1.8 m height and 80 kg weight. Figure 17A,B show a significant upward shift in the temperature ranges that are associated with heat risks for acclimatised vs. non acclimatized individuals. The comparison of Figure 17A,C shows the effect of adjusting clothing from light clothes (0.6 clo) to a cold weather attire (1.5 clo), which significantly reduces cold issues between 0 °C to 10 °C for moderate or higher activity levels.

### 4.4. Limitations

ClimApp is dependent on multiple sources to combine weather forecasting with personal factors to offer guidance on the assessment of heat and cold exposure and advice for short-term adaptation. Each information source may add error and influence the outcome of the ClimApp prediction. For instance, if the weather report is inaccurate, the ClimApp index is likely to be inaccurate as well. The personal context of the activity level and clothing level may also be poorly defined by users. Moreover, ClimApp does not consider the history of thermal exposure and assumes the user starts from a neutral condition. ClimApp does not take into account the effects of altitude or barometric pressure on heat balance as this is not included in the current ISO standards for heat and cold assessment. Therefore, ClimApp may not be suitable for use in high-altitude environments. Furthermore, in the present version of ClimApp, a 24 h forecast is provided to allow for the short-term planning of activities and decision making related to clothing, whereas a prolonged scheduling of work activities in occupational settings may require a longer time frame. However, this will also introduce increased uncertainty and we have therefore chosen to limit the forecasting period to a relatively short period (users and reader may see reference [4] for an example of a heat-warning platform with prolonged forecasting). Therefore, ClimApp, and any application of this type should be treated as a recommendation and should be used to gain insight in the short-term thermal stress conditions. ClimApp mitigates a portion of the inaccuracies by working with broad risk categories (e.g., heat risk levels are based on 20% blocks relative to WBGT thresholds). Nevertheless, the risk categorisations need further validation. Additionally, in outdoor conditions with potential exposure to a full solar load, ClimApp provides an analysis of the worst-case scenario. The user may therefore find that the observed situation is milder than ClimApp estimates, if they are staying in a partially shadowed area or if partial cloudiness reduces the radiative heat load. ClimApp is also bound by ISO human thermal models and their range of application. For instance, IREQ ISO 11079 claims to be valid between −50 °C and +10 °C; however, PHS ISO 7933 only starts from +15 °C to +50 C. Although field data suggest that IREQ can be stretched to +15 °C, there is no single ISO model that bridges the gap between cold and heat assessments [39]. ClimApp stretches the use of IREQ ISO 11079 by allowing it to operate until the WBGT thresholds are reached. The universal thermal climate index (UTCI) solves the gap in part and covers the entire temperature zone and is therefore also included in ClimApp’s forecast panel. However, the UTCI cannot be individualised and only assumes a moderate activity level (walking) with a clothing level based on the prevailing weather [13].

Another limitation to ClimApp is the current knowledge gap in the vulnerability and variability in subpopulations with respect to thresholds to define risk categories. For instance, for elderly people or children, the same threshold values are used as for adult workers, although the advice given is changed and is specifically provided to these two groups (see Figure 11). For elderly women, evidence shows that WBGT threshold values shift in warm conditions, but there is not yet sufficient evidence to warrant changes in algorithm modelling physiological functions [40]. Future thermophysiological studies are required to improve thermal risk assessments and generate action perspectives for vulnerable populations—including those that are not yet included in ClimApp (such as people with underlying diseases or using medication).

Finally, the present version of the app was developed within the ClimApp project period (see funding information in Acknowledgements section) and allows for future improvement and operation after the project period. On this basis and the developed framework, we believe that further developments and/or branching of the application are possible, e.g., targeting special populations or other advancements.

## 5. Conclusions

- The developed app has enabled the integration of weather forecasts with personal input to provide personalised heat and cold stress warnings.
- Users can customise their input by adjusting activity level, clothing, body characteristics, heat acclimatisation status, indoor or outdoor situation and manually change the geographical location if they wish to see forecasts for other locations than their current one (GPS determined).
- ClimApp supports the adaptation of appropriate thermoregulatory behaviour for a given warning by providing a weather summary, the thermal index and associated warnings of thermal stress, as well as advice in the form of infographics and text specific to the user profile.
- The app may support citizen science projects by letting users submit their local thermal environment conditions, clothing and activity levels, as well as their subjective responses.
- The research functionality and user evaluations are ongoing (currently with n = 1745) and have taken place across the globe in both warm and cold environments. From the present technical description and developed framework, the app may be versioned or further developed to target special populations for the advancement and benefit of user specificity.

## Figures and Tables

**Table 2:** Activity levels, description of activity level and corresponding heat production according to ISO8996.

Activity	Description	Q_met_ (W)
Low	Light manual work: writing, drawing, book-keeping. Easy to breathe and carry on a conversation	180
Moderate	Walking. Sustained arm and hand work: handling moderately heavy machinery, weeding, picking fruit	300
High	Intense arm and trunk work: carrying heavy material, shovelling, hand mowing, concrete block laying	415
Very high	Very intense occupational activity at fast maximum pace: working with an axe, climbing stairs, running on level surface.	520

**Table 3:** Clothing levels, description, clothing insulation value (I_cl_), vapour permeability index (im_st_), and air permeability (p_air_).

Clothing	Description	I_cl_	im_st_	p_air_
Summer attire	Loose-fitting, short clothing, typical for summer	0.078	0.45	200
Casual attire	Common in informal settings, long sleeves	0.124	0.38	100
Business suit *	Regular business suit, common in offices	0.155	0.38	50
Double layer	Generally taken to be coveralls over work clothes	0.233	0.38	10
Winter attire	Winter clothing, multiple layers including a thick coat	0.310	0.38	5
Extreme winter attire	Extreme cold weather clothing, multiple layers including a thick coat	0.388	0.38	5

* 0.155 m^2^KW^−1^ is equivalent to 1 Clo, corresponding to the insulation of a business suit.

## Data Availability

The source code can be found at https://github.com/ClimAppConsortium/ClimApp, accessed on 10 August 2021.
